# Metabolic syndrome: Nutri-epigenetic cause or consequence?

**DOI:** 10.1016/j.heliyon.2023.e21106

**Published:** 2023-10-17

**Authors:** Alfonso D. Silva-Ochoa, Erick Velasteguí, Isaac B. Falconí, Valeria I. García-Solorzano, Angie Rendón-Riofrio, Gabriela A. Sanguña-Soliz, Wim Vanden Berghe, Andrea Orellana-Manzano

**Affiliations:** a Laboratorio para Investigaciones Biomédicas, Facultad de Ciencias de la Vida, Escuela Superior Politécnica del Litoral, ESPOL, Campus Gustavo Galindo Km. 30.5 Vía Perimetral, P.O. Box 09-01-5863, Guayaquil, Ecuador; b Departamento de Ciencias de Alimentos y Biotecnología, Escuela Politécnica Nacional, Quito, Ecuador; c Escuela Superior Politécnica del Litoral, ESPOL, Centro de Agua y Desarrollo Sustentable, CADS, Campus Gustavo Galindo Km. 30.5 Vía Perimetral, P.O. Box 09-01-5863, Guayaquil, Ecuador; d Epigenetic signaling PPES lab, Department Biomedical Sciences, University Antwerp, Antwerp, Belgium; e Licenciatura en Nutrición y Dietética, Facultad de Ciencias de la Vida, Escuela Superior Politécnica del Litoral, ESPOL, Campus Gustavo Galindo Km. 30.5 Vía Perimetral, P.O. Box 09-01-5863, Guayaquil, Ecuador

**Keywords:** Nutrition, Epigenetics, Metabolic syndrome, Obesity, Diabetes

## Abstract

Metabolic syndrome is a cluster of conditions that results from the interplay of genetic and environmental factors, which increase the comorbidity risk of obesity, hyperglycemia, dyslipidemia, arterial hypertension, stroke, and cardiovascular disease. In this article, we review various high-impact studies which link epigenetics with metabolic syndrome by comparing each study population, methylation effects, and strengths and weaknesses of each research. We also discuss world statistical data on metabolic syndrome incidence in developing countries where the metabolic syndrome is common condition that has significant public health implications.

## Graphical abstract

**Figure:**
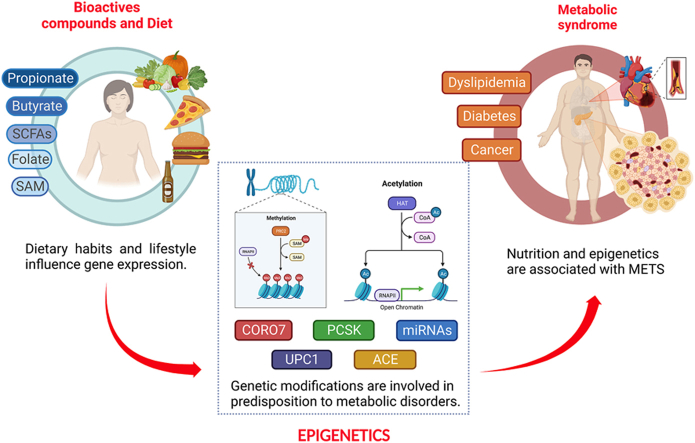

## Abbreviations

DNMT1: DNA methyltransferase 1
PWS: Prader-Willi syndrome
AS: Angelman syndrome
BWS: Beckwith-Wiedemann syndrome
RTT: Rett syndrome
ICF: Immunodeficiency, Centromere instability and Facial anomalies syndrome
DNMT3b: DNA Methyltransferase 3 Beta
MeCP2: Methyl-CpG binding protein 2
FTO: Fat mass and obesity
AT1R: Angiotensin type 1 receptor
RAAS: Renin-angiotensin-aldosterone system
IEAA: Intrinsic epigenetic age acceleration
EEAA: Extrinsic epigenetic age acceleration
HDL: High-density lipoprotein
BMI: Body mass index
ACE1: Angiotensin-converting enzyme 1
H3K9ac: Acetylation on histone H3 lysine 9
H3K9me3: Histone 3 lysine 9 trimethylation
H3K27me3: The histone 3 lysine 27 trimethylation
BP: Blood pressure
DM2: Type 2 diabetes mellitus
METS: Metabolic Syndrome
UCP1: Uncoupling protein 1
PCSK1 O PC1/3: Proprotein Convertase Subtilisin/Kexin Type 1
CORO7: Coronin7

## 1 Introduction

Metabolic syndrome is a complex group of conditions typically characterized by insulin resistance, or abdominal obesity, hyperglycemia, dyslipidemia, and arterial hypertension [1], which promotes chronic comorbidity diseases such as type II diabetes, cardiovascular diseases, and cancers or can elicit intergenerational epigenetic alterations in prenatal growth [2].

A low birth weight caused by malnutrition during pregnancy can also increase the risk of metabolic syndrome and cardiovascular disease in the offspring's adulthood, since metabolic rates are imprinted during embryogenesis, poor conditions during this period lead to the fetus acquiring a conservative metabolism that may not match with metabolic rates after birth when a sufficient diet is available [3,4]. Nutrition can also directly influence DNA methylation and development by changing methyl donor concentrations of S-adenosylmethionine [5]. Global hypomethylation has been shown to lead to chromosomal instabilities, such as rearrangements or translocations [6]. On the other hand, hypermethylation of CpG islands can lead to tumor suppressor gene silencing and predispose to cancer [6]. In addition, there are many non-hereditary genetic disorders, such as Prader-Willi syndrome (PWS), Angelman syndrome (AS), Beckwith-Wiedemann syndrome (BWS), Rett syndrome (RTT), and ICF syndrome, which are caused by genetic defects, causing epigenetic dysfunctions [7].

## 2 Nutrition and epigenetics

Epigenetics study how lifestyle conditions of individuals chemically modify genetic sequences and gene expression instructions through DNA methylation without changing the genetic code [8,9]. An illustration of the long-term impact of nutrition in epigenetics is the Dutch hunger winter in 1944 during the second world war. The Dutch women, including their neonates, suffered from an extreme undernutrition period of six months during pregnancy. Half a century later, children and grandchildren revealed twice the incidence of cardiovascular diseases, metabolic disorders, or cancer [10].

Malnutrition and starvation can also affect maternal and paternal imprinted genes, even with the programmed demethylation process.

GWAS studies have identified multiple genetic risk factors and structural variants involved in the development of metabolic diseases, a study in overweight children who exceeded adult weight (>80 kg) was found to fail to express leptin, a hormone that regulates the appetite [11]. This phenomenon was due to structural variants and not epigenetic modifications. Other studies revealed that people with two copies of the FTO gene have a higher body weight, around 3 kg more on average. The gene is linked to increased ghrelin levels [12]. Our genetic code is estimated to be responsible for between 40 and 70 % phenotypic variation in metabolic health [13], which environmental lifestyle factors can further modulate via an epigenetic mechanism. By studying how changes in gene expression, influenced by genetic modifications, may contribute to metabolic disorders, potencial advancements in the field could be made.

One of the most studied examples of medical relevance and history in epigenetics and nutrition occurred in 1944 during the “Hongerwinter” in Europe, particularly in the Netherlands. For a year, the German sociopolitical disputes deprived an entire country of food, causing thousands of deaths due to starvation [14]. This “starvation” generated a phenomenon of epigenetic change in those pregnant mothers who survived and transmitted a tendency to obesity to their offspring, particularly those who were already pregnant at the beginning of the famine [53,67]. This is due to the genome during embryogenesis and years later allows us to find a direct correlation between an individual's epigenome and their parents' lifestyle [11].

The Developmental Origins of Health and Disease (DOHaD) theory studies how early experiences and exposures, particularly during prenatal and early childhood, impact future health and disease. Environmental factors during critical development can program the organism, leading to long-term changes in structure, function, and gene expression. Research focuses on maternal nutrition, prenatal stress, chemical exposure, and intrauterine environment quality [68].

Epigenetic modifications are involved in phenotype transmission and predisposition to complex human diseases, including obesity and type 2 diabetes [15]. Methylation patterns can be inherited or influenced by the environment and can be highly stable. Recent studies showed that genetic variation and polymorphisms could also regulate DNA methylation changes in cis/trans via so-called methylation quantitative trait loci (mQTLs) [[16], [17], [18]]. DNA methylation in proximal promoter and enhancer regions has silencing effects on gene transcription. Meanwhile, DNA methylation in the gene body might stimulate transcriptional elongation and contribute to alternative splicing events [19].

Aging has been related to the onset of several chronic diseases due to cumulative epigenetic DNA methylation changes, and therefore, tools that estimate relative epigenetic aging speed have become very valuable as predictors of an individual's health status [20]. The main developed epigenetic clock models are Hannum's, Horvath's, and Weidner's [21]. However, some inconsistencies have been discovered between clock models when predicting the onset of various chronic diseases. This could be due to the model's limitation and tissue specificity indicating that DNA methylation age is not a universal health-disease marker [21]. Nevertheless, DNA methylation age may better estimate biological age than chronological age and may indirectly be a promising marker for health and disease status [22]. Although lifestyle factors, like stress and diet, impact the DNA methylation age, prolonged longitudinal studies in big cohorts of different ethnicities may be required to identify significant effects [21].

Lifelong environmental factors (e.g., salt intake, obesity, alcohol) and genetic factors contribute to the development of hypertension (Table 2). However, it has also been established that stress in utero may ‘program’ the later development of hypertension disease [24]. Angiotensin type 1 receptor (AT1R) plays a vital role in the renin-angiotensin-aldosterone system (RAAS) in blood pressure regulation [23]. A study with rats suggested that age and blood pressure affect CpG methylation in the promoter region of the AT1aR [24]. Systemic low-level inflammation is another common characteristic of older adults that may alter their response to infections [25].

## 3 Bioactive compounds in diet and epigenetics

**Fig. 1:**
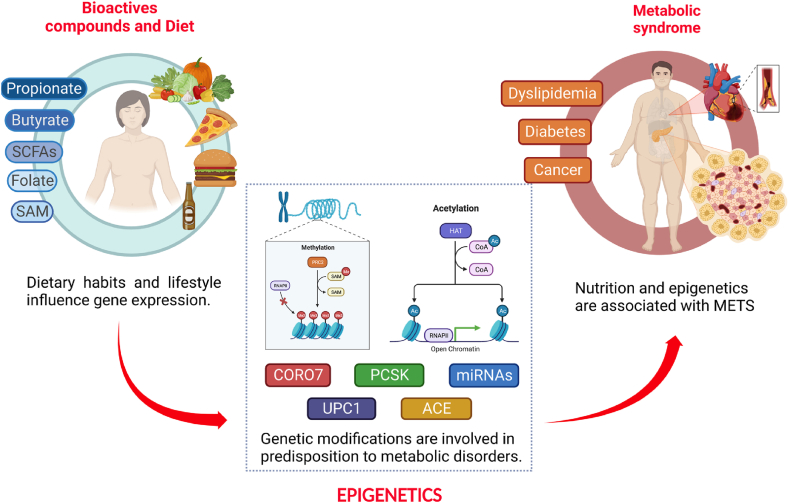
*Correlation between the bioactive compounds, diet, and epigenetics modification in metabolic syndrome.* The presence or absence of nutrients and bioactive compounds in the diet has been associated with epigenetic modifications in genes that regulate metabolic processes such as CORO7, PCSK, miRNAs, UPC1, and ACE. These genes' influence could lead to the predisposition to develop metabolic disorders and cancer.

Short-chain fatty acids (SCFAs) are among the main classes of bacterial metabolic products and are mainly synthesized in the colon through bacterial fermentation [54]. SCFAs mainly involve acetate, propionate, and butyrate (Fig. 1). Studies have shown that microbial metabolites, folate, B vitamins, and short-chain fatty acids interact with miRNAs to influence obesity phenotypes [55]. SCFAs generated by gastrointestinal microbiota significantly reduced resting angiotensin-converting enzyme 2 (ACE2) expression in cultured airway epithelial cells [56]. The oral administration of SCFAs in pigs can down-regulate the mRNA expressions of fatty acid synthase (FAS) and sterol regulatory element binding protein 1c and enhance the mRNA expression of carnitine palmitoyltransferase-1α (CPT-1α) in the liver. SCFAs can also decrease FAS, acetyl-CoA carboxylase (ACC), and peroxisome proliferator-activated receptor σ mRNA expressions in longissimus dorsi (add refs). In abdominal fat, SCFAs can reduce FAS and ACC mRNA expressions and increase CPT-1α mRNA expression [57].

Butyrate and propionate, produced in the intestine by the fermentation of dietary fiber, inhibit histone deacetylase enzymes, which increase histone acetylation and gene expression [58]. Butyrate indirectly regulates the activity of critical enzymes such as methylcytosine dioxygenase (TET) and DNA methyltransferase (DNMT1), thus modifying DNA methylation [59]. In addition, it can be oxidized to acetyl-CoA, therefore, it could increase histone acetylation, which occurs when an acetyl group is added to lysine residues in the N-terminal tails of histone proteins [60]. The presence or absence of nutrients and bioactive compounds in the diet is related to epigenetic modifications in genes that regulate metabolic processes such as CORO7, PCSK, miRNAs, UPC1, and ACE (Fig. 1). These genes' influence could lead to the predisposition to develop metabolic disorders and cancer.

During glycolysis, acetyl-CoA is derived from the catabolism of carbohydrates, lipids, and proteins in the mitochondria. Once formed, acetyl-CoA donates acetyl groups giving way to histone acetylation. This modification creates a more relaxed and open histone configuration, leading to the binding of transcription factors and RNA polymerase, increasing gene transcription levels [61].

The metabolite S-adenosylmethionine (SAM) is an essential methyldonor in cell differentiation and survival, regulating key metabolic pathways, including methylation and polyamine synthesis [62,63]. The excess of SAM catabolizes adenine and methylthioadenosine, which behave as toxic methylation inhibitors [63]. Table 1 provides some of the epigenetic studies performed in pacients with metabolic syndrome, methylation being specifically examined, the MetS component investigated, as well as the associated advantages and disadvantages of these studies.

**Table 1:** Relevant studies of epigenetics and metabolic syndrome.

Type of research	Sample	Methylation	MetS component	Advantages and positive sides	Disadvantages and limitations	References
Original research, observational, longitudinal	Humans, roughly 1000 underwent methylation	Intrinsic and extrinsic epigenetic age acceleration (IEAA and EEAA) were calculated from DNA methylation levels, it was showed that a greater number of MetS components is associated with more advanced epigenetic age acceleration	The more severe components in the Mets, the faster epigenetic acceleration is. Mets components: abdominal obesity, elevated triglyceride level, low HDL cholesterol levels, elevated blood pressure, or elevated fasting blood glucose levels.	Large human sample size	Population more than 60 years old were not included	[26]
Original research, observational, longitudinal	Humans, 4173 postmenopausal female participants from the Women's Health Initiative, as well as 402 male and female participants from the Italian cohort study, Invecchiare nel Chianti	DNA methylation age Intrinsic and Extrinsic Epigenetic Age Acceleration (IEAA, EEAA) were estimated.	Disturbances related to insulin and glucose, BMI and Waist-to-hip ratio, triglycerides and systolic blood pressure were related to an increase in epigenetic age acceleration, whereas HDL cholesterol, fish, fruits and vegetables, moderate alcohol, education, and exercise were related to a decrease in epigenetic age acceleration	Large human sample size	Inaccuracy of self-reported lifestyle habits, potential false negative results, differences in age, diet, culture, and other confounding factors as co-morbidities	[27]
Cross-sectional epigenome-wide association study	Humans, 648 individuals from the REgistre GIroni del COR (REGICOR) population-based cohort study for the discovery stage and 2568 participants from the Framingham Offspring Study's population-based cohort for the validation stage	70 CPGs related to obesity and 33 CPGs related to waist circumference were newly identified	BMI and waist circumference	Standardized methodology implemented to remove the non-biologic source of variation and the use of a large external population	The results present some heterogeneity and because it is a cross-sectional study, capacity to infer causality of the reported associations is limited	[28]
Original research, experimental	Male spontaneously hypertensive rats (SHRs) and age-matched Wistar-Kyoto (WKY) rats at three different postnatal ages corresponding to the pre-hypertensive (4 weeks), evolving (10 weeks), and established (20 weeks) stages of hypertension	Increased Angiotensin type 1 receptor AT1aR expression in SHRs is related to the AT1aR promoter hypo-methylation, which might be a consequence of the increased blood pressure and may be important in the maintenance of high blood pressure	Blood pressure	Good experimental design and grouping	It was not carried out in humans and the regulation of the AT1aR expression during the development of hypertension needs to be further elucidated	[24]
Original research, experimental	Eighteen-week–old Wistar-Kyoto (WKY) rats and SHRs	Angiotensin-converting enzyme 1 (ACE1) was differentially expressed in adrenal glands, heart, aorta, liver, lung and kidneys due to tissue-specific gene expression regulation	Blood pressure	Good experimental design and grouping	It was not carried out in humans and mechanisms were not elucidated	[29]
Original research, experimental	Humans, 60 healthy office workers and 60 truck drivers	Global H3K9ac, H3K9me3, and H3K27me3 levels measured in whole blood were negatively associated with pre-work BP measurements, H3K27me3 was positively associated with pulse pressure, but only among highly exposed truck workers	Blood pressure	Good human sample size	No gene was matched to methylation, no cellular or epigenetic mechanism were elucidated	[30]
Original research, experimental	Humans, 95 Finnish Caucasian patients (36 % males) were collected after fasting in the morning of a Roux-en-Y gastric bypass surgery from the Kuopio Obesity Surgery Study	In liver tissue, men and women had different methylation patterns, women had more methylation in X chromosomes whereas men had more methylation in autosomes, women had more methylation of *APLN* and *NKAP* genes; *XIST*, *KDM6A*, *ARSE*, and *RPS4X* from X chromosome, and *PKD2*, *H19*, and *PZP* from autosomes were more expressed in women than men; *VWCE*, *DGCR5*, *APOL2*, *PITPNM1*, *SDSL*, *FAM210B*, *SULT1A1* and *TTC39C* in autosomes were more expressed in men than women; *KDM6A* was more expressed in women than men was positively correlated with HDL-cholesterol thus representing a novel approach	HDL-cholesterol	Good human sample size and novelty	Uneven sample grouping, nonspecific probes from the HumanMethylation450 BeadChip array, patients had obesity	[31]
Original research, experimental	Female mice, divided in control or methyl-donor supplemented diet	Methyl-donors ameliorated development of atherosclerosis in offspring by inhibiting the T-cell Ccr2 expression (methylation), reducing inflammatory cytokines production and increasing serum HDL:LDL ratio; SR-B1, HMGCoR and PPAR-γ expression were increased and LDLr expression was decreased in offspring	HDL-cholesterol	Good experimental design	It was not carried out in humans	[32]
Original research, experimental	Humans, 11 participants without any metabolic condition and 34 metabolic patients, from which 25 participants were diagnosed with only T2D, and 9 participants were diagnosed with both MetS and T2D	No significant differences in *SCD1*, *PDK4*, *PDX1*, *FTO*, *KCNQ1*, *PPARg*, *PEG3*, and *KCNJ11* methylation between patients and controls were observed; differential methylation was observed between the groups in 4 single CpG loci located in the promoters regions of the genes *FTO*, *KCNJ11*, *PPARγ* and *PDK4*; a trend towards a positive correlation was observed for *PEG3* methylation with HDL cholesterol levels	HDL-cholesterol, blood glucose	Good experimental design, even grouping, age between 45 and 85 years	It was only measured between 3 and 9 CpG loci per gene and it is possible that other CpG loci located in the candidate genes are differently methylated between groups of participants, study population had a mean age of 70 years, it is well possible that larger differences in methylation levels between the different groups would have been observed in a younger population, where age-related decline of metabolic functions might not yet be as substantial, relatively small sample size (45 subjects)	[33]
Original research, genome-wide analysis, experimental	Humans, 119 Scandinavian men without known disease	It was identified genome-wide interactions between genetic and epigenetic variation in both *cis* and *trans* positions influencing gene expression in adipose tissue and in vivo (dys)metabolic traits associated with the development of obesity and diabetes	Cholesterol, HDL-cholesterol and fasting glucose	Human sample size, the study highlights the importance of genome-wide interactions between genetic and epigenetic variation and its role in human metabolism, they demonstrated for the first time an enrichment of significant mQTLs in adipose tissue on chromosome 6	Only a few phenotypes were considered it might require other phenotypes to discover all cause–effect relationships between SNPs, methylation and metabolic phenotype, more sophisticated analytical methods should be developed, relatively small sample size	[34]

**Table 2:** World statistics of metabolic syndrome.

Population & year of study	Index (%) of Metabolic Syndrome reported	Resume/Conclusions	Reference
EEUU (2018)	38.3 %	According to the US National Health and Nutrition Examination Survey (NHANES) 2011–2018, the prevalence of MetS during the years analyzed was constant, but an increase of it on the non-Hispanic Asians occurred, from 21.8 % to 31.2 %.	[37]
Canada (2019)	Adults (20–79 y/o): Prediabetes (12.4 %), Diabetes (7-5%), Undiagnosed diabetes (37.3 %).	The intention of this study was to evaluate and compare the different eating habits in between adults with diagnosed diabetes, pre diabetes and healthy adults. Undiagnosed adults had a higher intake of sugar-contaning foods, while diagnosed adults had a diet rich in carbohydrates.	[38]
Arabia (2014)	Adults (15–64 y/o): 28.3 %	Industrialization and accessibility to fast foods have caused a rise of MetS in Saudi Arabia. Prevalence was higher in males than in females.	[39]
Sub-Saharan Africa (2020)	Adults: IDF (18.0 %), IDF-ethnic (16.0 %), JIS (23.9), NCEP-ATP III (17.1 %), WHO (11.1 %).	Metabolic syndrome diseases have been on the rise in Sub-Saharan African countries. A systematic analysis was done in order to estimate the prevalence of Metabolic Syndrome in adults according to different criteria. It was concluded that women and the population in urban areas were most affected**.**	[40]
Global (2020)	Children (6–12 y/o): Nicaragua (5.2 %), Iran (8.8 %), Mexico (12.3 %), Northwestern Europe (1.4 %), Central Latin America (8.2 %). Adolescents (13 - 18y/o): East Asia (2.9 %), Iran (9.0 %), United Arab Emirates (9.8 %), Spain (9.9 %), high-income English-speaking countries (6.7 %)	In this study, the prevalence of Metabolic Syndrome in children and adolescentes did not show a correlation with the country's developmental level. And it estimates that globally, the frequency of metabolic syndrome in children and adolescents is 2.8 % and 4.8 %, respectively.	[41]
Ecuador (2021)	Adults: 18 - 59 y/o: 31.2 % women: 30.8 % men: 31.5 %	Ecuador's studied population was divided by sex, ethnic origin, urban or rural location, region, altitud and economic status. Data obtained in the ENSANUT - ECU 2012 shows that MetS prevalence has no significant difference between genders, that the 50–59 y/o group has the highest incidence of his disorder as well as mestizos, urban, 0–500 mASL. and Coast and Galapagos population. This study also hypothesizes that the higher the socioeconomic class, the lower the prevalence of MetS in the population.	[42]
Middle Eastern Countries (2009)	Adults: (25–64 y/o) ATP III Criteria: 34.7 % IDF Definition: 37.4 % ATP III/AHA/NHLBI criteria: 41.6 %	The definitions of MetS correspond to the Adult Treatment Panel III (ATP III), the International Diabetes Federation (IDF), the American Heart Association (AHA) and the National Heart, Lung, and Blood Institute (NHLBI). In this study, data obtained from Iran, Turkey, Oman and Tunisia showed that the 55–64 y/o population present a higher prevalence of MetS, and women in urban areas are the population with the highest estimations of this disorder.	[43]
Central America (2015)	Adults: (20–65 y/o) According to ATP III Criteria: 30.3 % Honduras: 23.0 % Costa Rica: 35.1 % Belize: 32 % Guatemala: 31.6 % Nicaragua: 30 %	The 2 countries with the highest MetS and diabetes prevalence are Belize and Costa Rica. The data collected showed that the 20–39 y/o age group, women and those without a paid job population have the highest frequency of MetS. And generally, the prevalence of this disorder is higher in Central America than in Mexico or other more developed countries.	[44]
Korea (2020)	Adults: Men: 28.1 % Women: 18.7 %	The Korea National Health and Nutrition Examination Survey (2008–2017) provided the information. In the analysis of the data collected, there is a significant relationship between the prevalence of MedS and men with obesity, smoking and drinking habits. Meanwhile for women, the higher risk belongs to the population with obesity and smoking habits. It also shows that the risk of presenting MedS decreases as the economical level and incomes increase.	[45]

## 4 Population genetics and metabolic disorders

The “thrifty gene” hypothesis suggested that people predisposed to obesity and type 2 diabetes might belong to a human subgroup more adapted to storing nutrients, increasing their chances of surviving during a famine [35]. Over the past six decades, extensive GWAS studies have established an undeniable relationship between an individual's metabolic disorder and genetic makeup [36]. Evaluating the global incidence of metabolic syndrome presents a complex challenge due to the diverse social and economic factors that impact the nutritional status of populations. Enclosed below is a listing of countries and their corresponding MetS prevalence index. Table 2 summarizes the global statistics in various metabolic syndrome categories and age groups, along with a brief description of each study approach.

The correlation between the economic level and the prevalence of Metabolic Syndrome in the listed countries is robust. It has been observed that areas with lower incomes are at a higher risk of developing the cluster of symptoms associated with this disorder (Table 2). This provides insight into the dietary habits of each region. Interestingly, while developed countries have a higher incidence of obesity, less developed regions and countries are at a greater risk of Metabolic Syndrome.

## 5 Epigenetic inheritance

Increasing evidence indicates that non-DNA sequence-based epigenetic information can be inherited across several generations in organisms ranging from yeast to plants to humans. This raises the possibility of heritable ‘epimutations’ contributing to heritable phenotypic variation and, thus, to evolution [64]. Transgenerational epigenetic changes induced by hypoxia can result in permanent changes early in fetal development [66]. For instance, polyphenols can inhibit endothelial dysfunction when considering dyslipidemias at the molecular level because they reduce oxidative stress and increase Nitric Oxide (NO) production [65]. Dietary polyphenols are key in modulating epigenetic-sensitive mechanisms involved in vascular endothelium homeostasis. An example is revestratol, a polyphenol usually found in diets via fruits and vegetables. Revestratol influences the activity of histone-modifying enzymes and DNA methyltransferases, contributing to epigenetic modifications [69].

Interestingly, a pleiotropic SNP (rs964184) harbored in the ZPR1 zinc finger (ZNF259) gene resulted in *cis*-associated with the expression of the proprotein convertase subtilisin/kexin type 7 (PCSK7) gene promoting the interindividual variation in LDL-C, HDL-C, and TAG plasma levels suggesting a novel therapeutic target, Table 1. Despite the increasing knowledge on lipidome-related molecular perturbations at early and late stages of life and how the infant can keep dysregulated epigenetics marks established during that time and alter their lipid metabolism [70], current risk assessment and pharmacological management of dyslipidemias are not satisfying [66].

## 6 Genes linked to metabolic disorder

### 6.1 ACE

Recent research has indicated that the ACE gene could notably impact METS, mainly when regulating blood pressure during exercise and releasing Nitric Oxide (NO), Table 1. It has been discovered that the insertion/deletion polymorphism (rs4646994) of this gene has a strong correlation with a reduction in NO release, lower hypertension rates, and increased levels of angiotensin-converting enzyme [46].

### 6.2 UCP1

Research on the Saudi population has found that the UCP1 gene plays a significant role in energy metabolism and is linked to obesity, Table 1. Two variations, known as rs1800592 and rs3811791, have been associated with moderate obesity and affect the availability of functional proteins, impacting oxidative phosphorylation and energy expenditure [47].

### 6.3 PCSK1

Proprotein Convertase Subtilisin/Kexin Type 1 (PCSK1 or PC1/3) has been associated with obesity, body mass index, birth weight, and proinsulin levels [50]. Rare mutations in PCSK1 have also been implicated in early monogenic obesity. Null mutations in the PCSK1 gene can cause morbid obesity, hypoadrenalism, hypogonadism, bowel dysfunction, hyperphagia, impaired proinsulin-insulin ratio, postprandial hypoglycemia, and diabetes insipidus [48]. A deficiency in PC1/3 activity has severe gastrointestinal consequences from birth, including recurrent watery diarrhea, weight loss, dehydration, and metabolic acidosis. In addition, they resulted in hospitalization and parenteral nutrition, Table 1.

In certain instances, children may pass away during their early years. Although intestinal biopsies indicate no visible abnormalities, there is a notable failure to absorb fats and amino acids. Despite the intestine's structural soundness and preserved villous architecture, this lack of absorption is severe [49].

### 6.4 CORO7

The human gene Coronin7 (CORO7 or CRN7) acts as a POD1 analog and regulates metabolic balance and body weight by controlling the central feeding circuits, CpG islands near the CORO7 promoters exhibit lower methylation in overweight children, leading to higher CORO7 expression [50]. In contrast, in rats, reduced food intake resulted in decreased expression of this gene, which is associated with decreased appetite stimulation [50]. The relationship between dietary patterns and the expression of CORO7 was observed in regions of the brain responsible for regulating energy balance, such as the hypothalamus, which is particularly sensitive to feeding behaviors [51].

## 7 Conclusions

Susceptibility to metabolic disorder, in part, is determined by an individual's genome configuration, which hosts MetS risk alleles and/or SNPs.

New studies have also identified the significant complementary contribution of environmental lifestyle factors, which further propagate MetS risk via epigenetic DNA methylation silencing mechanisms. However, further research is required to untangle the genetic-epigenetic crosstalk in MetS. The high costs of investigating allelic variants and an individual's epigenome in big cohort studies pose a logistic socioeconomic challenge in developing countries with an increased incidence of malnutrition. New, cost-effective 4th-generation sequencing technologies may create new opportunities for the combined identification of allelic variants in long sequences and methylated cytokines in epigenetics without bisulfite conversion [52].

Population (epi)genetics studies can provide valuable insights into metabolic disorders, their prevalence, and their potential impact on future generations. By identifying epigenetic variants within a population, public health and prevention systems can be tailored to improve the quality of life and reduce the costs associated with treating metabolic disorders. Given the unique nutritional needs and epigenetic effects of different countries and demographics, conducting these studies within each population is essential.

## Author contribution statement

All authors listed have significantly contributed to this article's development and writing.

## Data availability statement

Data included in article/supp. Material/referenced in article.

## Declaration of competing interest

The authors declare that they have no known competing financial interests or personal relationships that could have appeared to influence the work reported in this paper.
